# *Isospora similisi* recovered from a new host, *Saltator aurantiirostris*, with supplementary molecular data and notes on its taxonomy and distribution in the Neotropical region

**DOI:** 10.1590/S1984-29612024060

**Published:** 2024-10-07

**Authors:** Carla Maronezi, Carlos Nei Ortúzar-Ferreira, Mariana de Souza Oliveira, Patrícia Barizon Cepeda, Viviane Moreira de Lima, Bruno Pereira Berto

**Affiliations:** 1 Programa de Pós-graduação em Ciência, Tecnologia e Inovação em Agropecuária, Universidade Federal Rural do Rio de Janeiro – UFRRJ, Seropédica, RJ, Brasil; 2 Programa de Pós-graduação em Biologia Animal, Instituto de Ciências Biológicas e da Saúde, Universidade Federal Rural do Rio de Janeiro – UFRRJ, Seropédica, RJ, Brasil; 3 Centro Universitário de Barra Mansa, Barra Mansa, RJ, Brasil; 4 Departamento de Biologia Animal, Instituto de Ciências Biológicas e da Saúde, Universidade Federal Rural do Rio de Janeiro – UFRRJ, Seropédica, RJ, Brasil

**Keywords:** Coccidia, oocysts, taxonomy, sequencing, phylogeny, Itatiaia National Park, Coccídios, oocistos, taxonomia, sequenciamento, filogenia, Parque Nacional de Itatiaia

## Abstract

This article reports on a golden-billed saltator *Saltator aurantiirostris* Vieillot, 1817, kept in captivity outside its natural distribution area, in the proximities of the Itatiaia National Park, as a new host for *Isospora similisi* Coelho, Berto, Neves, Oliveira, Flausino & Lopes, 2013. Additionally, a supplementary molecular identification is provided through the sequencing of three non-overlapping *loci* of mitochondrial DNA and one *locus* of the 18S small subunit ribosomal RNA (18S) gene. All the taxonomic features of the *I. similisi* oocysts shed by *S. aurantiirostris* were equivalent to those originally described from *Saltator similis* d’Orbigny & Lafresnaye, 1837. The new sequenced *loci* were identical, or showed 99.9% similarity, to the samples of *I. similisi* from *S. similis* and *S. aurantiirostris*, confirming the same species from both hosts. Lastly, *I. similisi* is estimated as a junior synonym of *Isospora formarum* McQuistion & Capparella, 1992, due to the morphological similarities and wide distribution of its hosts in the Neotropical region. Therefore, this study encourages future taxonomic inquiries into *I. similisi* collected from other *Saltator* spp. in order to establish this synonymization of *I. formarum* with *I. similisi*, and hence, its wide distribution and dispersion in the Neotropical region, including across the Andes mountains.

## Resumo

Este artigo relata um bico-duro *Saltator aurantiirostris* Vieillot, 1817, mantido em cativeiro fora de sua área de distribuição natural, nas proximidades do Parque Nacional de Itatiaia, como novo hospedeiro de *Isospora similisi* Coelho, Berto, Neves, Oliveira, Flausino & Lopes, 2013. Adicionalmente, uma identificação molecular suplementar foi fornecida por intermédio do sequenciamento de três *loci* não sobrepostos de DNA mitocondrial e um *locus* da subunidade pequena do gene 18S do RNA ribossômico (18S). Todas os caracteres taxonômicos dos oocistos de *I. similisi,* eliminados por *S. aurantiirostris,* foram equivalentes àqueles originalmente descritos de *Saltator similis* d'Orbigny & Lafresnaye, 1837. Os novos *loci* sequenciados foram idênticos, ou tiveram 99,9% de similaridade, com as amostras de *I. similisi* de *S. similis* e *S. aurantiirostris*, confirmando a mesma espécie de ambos os hospedeiros. Por fim, *I. similisi* foi estimada como sinonímia júnior de *Isospora formarum* McQuistion & Capparella, 1992, devido às semelhanças morfológicas e ampla distribuição de seus hospedeiros na região Neotropical. Portanto, este estudo incentiva futuras investigações taxonômicas sobre *I. similisi,* recuperados de outros *Saltator* spp., a fim de estabelecer essa sinonimização de *I. formaru*m com *I. similisi* e, consequentemente, sua ampla distribuição e dispersão na região Neotropical, inclusive Cordilheira dos Andes.

## Introduction

Saltators (*Saltator* spp.) are passerines of the family Thraupidae and subfamily Saltatorinae with a diversity of 19 species and an exclusively Neotropical distribution. Of this total, only nine *Saltator* spp. occur in Brazil (Chaves et al., 2013; BirdLife International, 2024). The green-winged saltator *Saltator similis* d’Orbigny & Lafresnaye, 1837 is the most well-known and valued *Saltator* sp. in Brazil for breeding in captivity, due to its vocal repertoire, which is why it is one of the main wild bird species illegally captured and trafficked. The golden-billed saltator *Saltator aurantiirostris* Vieillot, 1817, in turn, is a passerine uncommonly observed in captivity and rarely related to animal trafficking in Brazil, despite also having a vocal repertoire typical of the Saltarorinae (Nunes et al., 2012; Freitas et al., 2015).

Coccidiosis stands out among the parasitic diseases of passerines, including *Saltator* spp. There have been a few reports of symptomatic and/or severe coccidiosis in captive green-winged saltators and buff-throated saltators *Saltator maximus* (Müller, 1776) (Coelho et al., 2012; Vasconcellos et al., 2013; Maronezi et al., 2022), and also in the wild (Maronezi et al., 2024). However, scientific literature contains no reports of coccidians collected from other *Saltator* spp. in Brazil, which are wild species difficult to capture and/or less valued for commercial breeding in captivity (Nunes et al., 2012; Freitas et al., 2015; Maronezi et al., 2022).

In this context, the aim of the current study was to record a golden-billed saltator *S. aurantiirostris*, living in captivity outside its natural distribution area, in the municipality of Resende, in the proximities of Itatiaia National Park (Parque Nacional de Itatiaia) in the state of Rio de Janeiro, as a new host for *Isospora similisi* Coelho, Berto, Neves, Oliveira, Flausino & Lopes, 2013, which is a coccidian species so far recorded only on green-winged saltators *S. similis*. In addition, a supplementary molecular identification is provided through the sequencing of three non-overlapping *loci* of mitochondrial DNA and one *locus* of the 18S small subunit ribosomal RNA (18S) gene. Also included are taxonomic notes on the strong evidence of synonyms between *Isospora* spp. recorded from *Saltator* spp., based on the current new record, morphological equivalence and the wide distribution areas of host *Saltator* spp. in the Neotropical region.

## Materials and Methods

### Sample collection

Fecal samples were collected from an adult male specimen of golden-billed saltator *S. aurantiirostris* owned by a breeder located near the Itatiaia National Park (22°28’0”S, 44°27’56”W), which includes part of the municipalities of Itatiaia and Resende in the state of Rio de Janeiro, southeastern Brazil. This park is a Brazilian federal conservation unit with an extremely high priority for biodiversity conservation (ICMBIO, 2016). The golden-billed saltator was already banded with metal ring associated with the Registration System of Passeriformes (Sistema de Cadastramento de Passeriformes - SISPASS) of the Brazilian Institute for the Environment and Renewable Natural Resources (Instituto Brasileiro do Meio Ambiente e dos Recursos Naturais Renováveis - IBAMA), which, in principle, confers an origin of legalized captive breeding; however, the life history of this passerine was inaccessible and unknown to the owner who claimed to have recently received the passerine as a donation. The bottom of the cage where the golden-billed saltator was kept was lined with clean paper for a period of 5 hours (2 pm to 7 pm) to collect its droppings. During this period, the paper was examined and replaced several times in order to obtain fresh fecal droplets separately. Each fecal droplet was placed individually in a centrifuge tube with a solution of 2.5% potassium dichromate (K_2_Cr_2_O_7_) (Dolnik, 2006).

### Morphological analysis

Samples were examined at the Laboratory of Coccidia Biology (Laboratório de Biologia de Coccídios - LABICOC) of the Federal Rural University of Rio de Janeiro (Universidade Federal Rural do Rio de Janeiro - UFRRJ). All the samples were incubated at room temperature (25 °C) for 7 days. Oocysts were isolated by flotation in Sheather’s sugar saturated solution (specific gravity: 1.20) (Duszynski & Wilber, 1997). Sample density was calculated as the number of oocysts per fecal droplet (Dolnik, 2006; Bush et al., 1997). Morphological observations and measurements were taken following the guidelines of Duszynski & Wilber (1997) and Berto et al. (2014a), using an Olympus BX binocular microscope (Olympus Optical, Tokyo, Japan) equipped with a Eurekam 5.0 digital camera (BEL Photonics, Monza, Italy). Photomicrographs and other figures were edited using two software programs (Corel DRAW and Corel PHOTO-PAINT) from CorelDRAW® (Corel Draw Graphics Suite, Version 2020, Corel Corporation, Canada). All the measurements are shown in micrometers and are given as the range, followed by the mean in parentheses.

### Obtaining representative specimens of *Isospora similisi*

Some of the representative specimens of *I. similisi* collected from *S. similis* identified in Maronezi et al. (2022), which are deposited in the Parasitology Collection of the LABICOC (UFRRJ, 2024) at UFRRJ under Repository No. 118/2021, were requested for supplementary molecular identification and comparison.

### Molecular analysis

Individual oocysts morphologically identified as *I. similisi*, both from the original collection (representative specimens of *S. similis*) deposited by Maronezi et al. (2022), and from *S. aurantiirostris* of the current study, were photomicrographed under light microscopy, isolated, resuspended in 0.9% phosphate buffered saline (PBS), and washed by centrifuging until the supernatant became clear (Dolnik et al., 2009). DNA was extracted from the individual oocysts, using a Quick-DNA Micropep kit from Zymo Research Corporation according to the manufacturer’s instructions. Four freeze-thaw cycles were applied prior to DNA extraction in order to ensure complete lysis of the oocysts. PCR amplification was performed for three non-overlapping *loci* in *cox1* (MAVCOXI), *cox3* (MACOIII) genes and fragments of small and large subunit rDNA (MARI) of mitochondrial DNA (Ortúzar-Ferreira et al., 2023), and one *locus* of the 18S small subunit ribosomal RNA (1NF/18S) (Andrade et al., 2024). For amplification, a 25 µl PCR reaction was prepared using 3 µl of genomic DNA (<1 µg), 12.5 µl of GoTaq® G2 Hot Start Colorless Master Mix (Promega Labs) (1X), 0.25 µl of each Primer (0.2 µM) and 9µL of Nuclease Free Water. PCR amplifications were conducted using the cycling conditions originally described by Ortúzar-Ferreira et al. (2023) and Andrade et al. (2024).

### DNA sequence analysis

All the PCR products were sequenced with PCR forward and reverse primers by Ludwig Biotechnology, using an ABI-Prism 3500 Genetic Analyzer (Applied Biosystems, Foster City, California) for Sanger sequencing. The results of the sequencing reactions were analyzed and edited in the Chromas 2.6 program. Sequences were compared with other eimeriid coccidia available in the GenBank database, using the Basic Local Alignment Search Tool (BLAST). Alignments were created in MEGA v10.2.6 using Clustal W. Phylogenetic relationships were reconstructed using Bayesian Inference in MrBayes v3.2.7 software (Ronquist et al., 2012) and the Maximum likelihood method in the MEGA software (Kumar et al., 2018). The evolutionary model that best fit all the phylogenetic analyses was selected using the Model Selection tool of MEGA software. Bayesian Inference analysis was conducted under the GTR+G evolutionary model for 1,000,000 generations, and the trees were summarized after removing 25% of burn-in. Maximum likelihood analysis was conducted under the TN93+G evolutionary model, and the bootstrap values were calculated using 1,000 replicates. The resulting phylogenetic trees were viewed in the MrBayes and MEGA software programs and exported in FigTree v1.4.4.

## Results

### Examination of samples and identification of species

Four samples (fecal droplets) from the golden-billed saltator *S. aurantiirostris* were examined and found positive for coccidian oocysts, which were morphologically identified as *I. similisi*. The taxonomic features observed are described below:

### *Isospora similisi* Coelho, Berto, Neves, Oliveira, Flausino & Lopes, 2013 (Figure 1)

**Figure 1:**
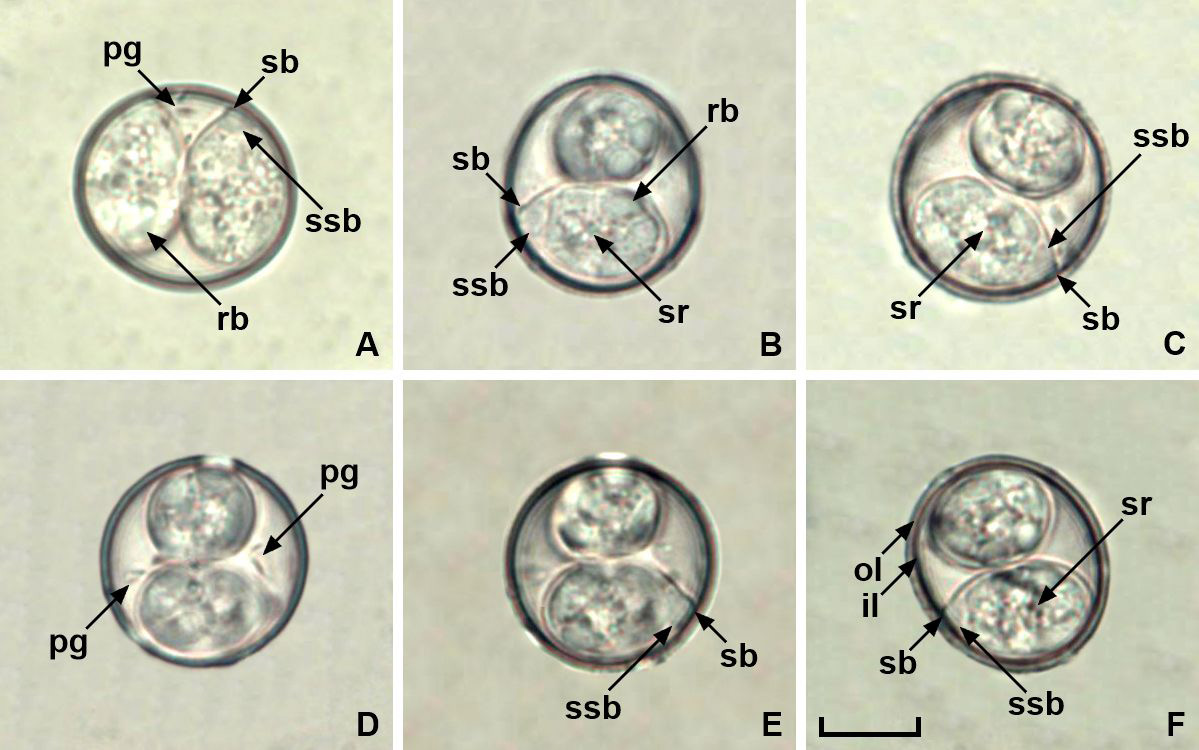
Photomicrographs of sporulated oocysts of *Isospora similisi* from a golden-billed saltator *Saltator aurantiirostris,* highlighting the main taxonomic characters (A-F). Note the inner (il) and outer (ol) layers of the oocyst wall; polar granules (pg); Stieda (sb) and sub-Stieda bodies (ssb); sporocyst residuum (sr); and refractile body (rb). Scale-bar: 10 µm.

Oocyst (n = 22) subspherical to ovoid, 20-25 × 20-22 (22.8 × 21.3); length/width (L/W) ratio 1.0-1.2 (1.09). Wall bi-layered, 1.3-2.0 (1.6) thick, outer layer smooth, c.2/3 of total thickness. Micropyle and oocyst residuum are absent, but splinter-like or comma-like polar granules are present. Sporocyst (n = 16) ovoid to slightly piriform, 14-17 × 10-11 (15.7 × 10.6); L/W ratio 1.4-1.6 (1.50). Stieda body present, half-moon-shaped to knob-like, 0.8-1.2 high × 2.0-2.4 wide (1.0 × 2.2). Sub-Stieda body present, rounded to trapezoidal, 1.5-2.4 high × 3.9-4.4 wide (2.0 × 4.1), frequently with density variations resembling a compartmentalized sub-Stieda. Para-Stieda body absent. Sporocyst residuum present, consisting of spherules scattered among the sporozoites. Sporozoites vermiform, with posterior refractile body and a central nucleus.

### Taxonomic summary

Type host: *Saltator similis* d’Orbigny & Lafresnaye, 1837 (Aves: Passeriformes: Thraupidae: Saltatorinae), green-winged saltator (Coelho et al., 2013; Maronezi et al., 2022, 2024).

Other host: *Saltator aurantiirostris* Vieillot, 1817 (Aves: Passeriformes: Thraupidae: Saltatorinae), golden-billed saltator (current study).

Type locality: Centro de Triagem de Animais Silvestres – CETAS (Wild Animal Screening Center) in the municipality of Seropédica, state of Rio de Janeiro, southeastern Brazil (22°43’24”S, 43°42’37”W) (Coelho et al., 2013).

Other localities: Five sites of captivity in the proximities of Itatiaia National Park (22°30’S, 44°34’W), southeastern Brazil (Maronezi et al., 2022). Kilometer 5 of the ‘Travessia Ruy Braga’ (Ruy Braga Crossing) in Itatiaia National Park (free-living *S. similis* captured) (22°25’39.9”S; 44°37’49.4”W), southeastern Brazil (Maronezi et al., 2024). Site of captivity in the municipality of Resende, adjacent to Itatiaia National Park, in the state of Rio de Janeiro (22°28’0”S, 44°27’56”W), southeastern Brazil (current study).

Type-material: Photos of syntypes and line drawings are deposited and available (UFRRJ, 2024) in the Parasitology Collection of the Laboratory of Coccidia Biology at UFRRJ, under Repository No. P-41/2011 (Coelho et al., 2013). Physical material (preserved oocysts) has been lost or is unavailable.

Representative specimens: Photomicrographs, line drawing and oocysts in 2.5% K_2_Cr_2_O_7_ solution are deposited and available (UFRRJ, 2024) in the Parasitology Collection of the Laboratory of Coccidia Biology at UFRRJ, under Repository Nos. 118/2021 (Maronezi et al., 2022) and 137/2024 (current study). Photographs of the host specimens are deposited in the same collection.

Representative DNA sequences: DNA amplification of the oocysts from *S. similis* by Maronezi et al. (2022) and *S. aurantiirostris* in the current study, at the MAVCOXI, MACOIII, MARI and 1NF/18S *loci*, showed clear bands around ~653 bp, ~632 bp, ~824 bp and ~444 bp, respectively. Representative sequences are deposited in the GenBank database under Accession Nos. PP723065 (MAVCOXI, *S. similis*); PP723066 (MACOIII, *S. similis*); PP723067 (MACOIII, *S. aurantiirostris*); PP723068 (MARI, *S. similis*); PP723069 (MARI, *S. aurantiirostris*); PP716360 (1NF/18S; *S. similis*); and PP716361 (1NF/18S; *S. aurantiirostris*).

Site of infection: Unknown, oocysts were recovered from feces.

Prevalence: 100% (1/1).

Density: Mean of 5,786 (ranging from 3,358 to 9,000) oocysts in 4 fecal droplets collected from the golden-billed saltator *S. aurantiirostris* during the 3-hour period of 2 pm to 7 pm.

### Molecular analysis

The sequences at the four gene *loci* obtained from oocysts morphologically identified as *I. similisi* differed by several nucleotides when compared with sequences from eimeriid coccidians deposited in GenBank. The sequences from the oocysts of *I. similisi* recovered from *S. similis* by Maronezi et al. (2022), in comparison with those obtained from *S. aurantiirostris*, were identical (1NF/18S) or showed 99.9% similarity (MACOIII and MARI), depending on the *locus* analyzed.

Only the representative specimens of *I. similisi* from *S. similis* recovered from the collection deposit amplified in the MAVCOXI *locus*; therefore, it was not possible to compare them with samples from *S. aurantiirostris* for this *locus*. In any case, this sequence for the MAVCOXI *locus* of *I. similisi* was maintained and phylogenetically analyzed in the current study, as it is unprecedented for any host. In comparison with sequences deposited in GenBank, *I. similisi* showed the highest similarities, i.e., approximately 98%, to *Isospora* spp. collected from superb starlings *Lamprotornis superbus* Rüppell, 1845 in Canada (Hafeez & Barta, 2017).

In the MACOIII *locus*, the sequences of *I. similisi* from *S. similis* and *S. aurantiirostris* were dissimilar in the substitution of only two nucleotides. In GenBank, these sequences were approximately 98% similar to *Isospora* spp. described/reported from island canaries *Serinus canaria* (Linnaeus, 1758) (Ogedengbe et al., 2016), superb starlings *L. superbus* (Hafeez & Barta, 2017) and indigo buntings *Passerina cyanea* (Linnaeus, 1766) (unpublished data; GenBank Accession No. MW645337).

In the MARI *locus*, a comparison of *I. similisi* from the two hosts indicated that the sequences differed by a single nucleotide. In GenBank, the highest similarities between *Isospora* spp. described/reported from *S. canaria* (Ogedengbe et al., 2016) and *L. superbus* (Hafeez & Barta, 2017) in this *locus* were approximately 99%.

The sequences of *I. similisi* from the two hosts were identical in the 1NF/18S *locus*, and showed the highest similarities, i.e., 99.7%, with *Isospora* spp. described/reported from red-browed finches *Neochmia temporalis* (Latham, 1801) (Yang et al., 2016), superb starlings *L. superbus* (Hafeez & Barta, 2017), common starlings *Sturnus vulgaris* Linnaeus, 1758 (unpublished data; GenBank Accession No. MW667591), yellow-shafted flickers *Colaptes auratus* (Linnaeus, 1758) (unpublished data; GenBank Accession No. MW618926), rock doves *Columba livia* Gmelin, 1789 (Matsubara et al., 2017) and austral thrushes *Turdus falcklandii* Quoy & Gaimard, 1824 (Martínez et al., 2015).

### Phylogenetic analysis

**Figure 2:**
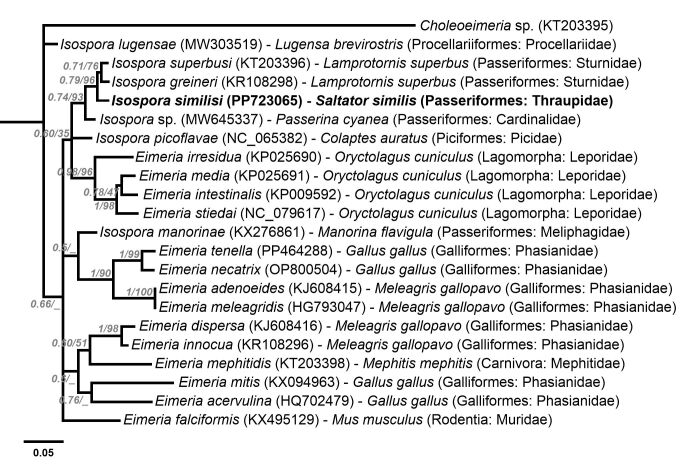
Phylogenetic relationship of *Isospora similisi* from the green-winged saltator *Saltator similis* inferred by Bayesian analysis for a *locus* (MAVCOXI) within *cox1* gene of the mitochondrial genome. Branch lengths correspond to mean posterior estimates of evolutionary distances (scale-bar: 0.05). Branch labels at the nodes show posterior probabilities under the Bayesian Inference analysis and bootstrap values derived from Maximum likelihood analysis. Only posterior probabilities higher than 0.5 are displayed. The phylograms were outgrouped using an unnamed *Choleoeimeria* sp. (GenBank accession number: KT203395).

**Figure 3:**
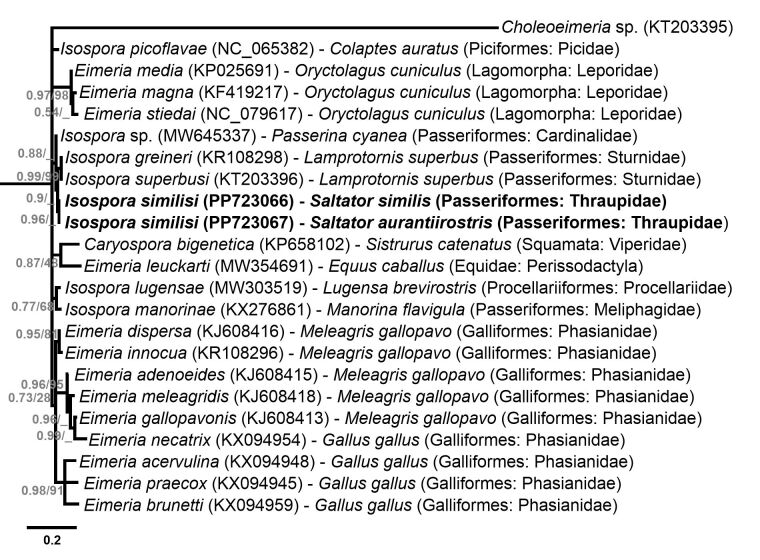
Phylogenetic relationship of *Isospora similisi* from the green-winged saltator *Saltator similis* and golden-billed saltator *Saltator aurantiirostris* inferred by Bayesian analysis for a *locus* (MACOIII) within *cox3* gene of the mitochondrial genome. Branch lengths correspond to mean posterior estimates of evolutionary distances (scale-bar: 0.2). Branch labels at the nodes show posterior probabilities under the Bayesian Inference analysis and bootstrap values derived from Maximum likelihood analysis. Only posterior probabilities higher than 0.5 are displayed. The phylograms were outgrouped using an unnamed *Choleoeimeria* sp. (GenBank accession number: KT203395).

**Figure 4:**
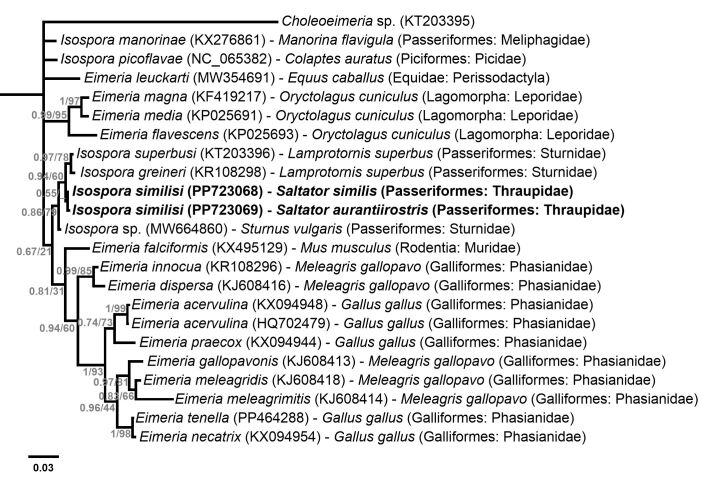
Phylogenetic relationship of *Isospora similisi* from the green-winged saltator *Saltator similis* and golden-billed saltator *Saltator aurantiirostris* inferred by Bayesian analysis for a *locus* (MARI) within fragments of small and large subunit rDNA of the mitochondrial genome. Branch lengths correspond to mean posterior estimates of evolutionary distances (scale-bar: 0.03). Branch labels at the nodes show posterior probabilities under the Bayesian Inference analysis and bootstrap values derived from Maximum likelihood analysis. Only posterior probabilities higher than 0.5 are displayed. The phylograms were outgrouped using an unnamed *Choleoeimeria* sp. (GenBank accession number: KT203395).

**Figure 5:**
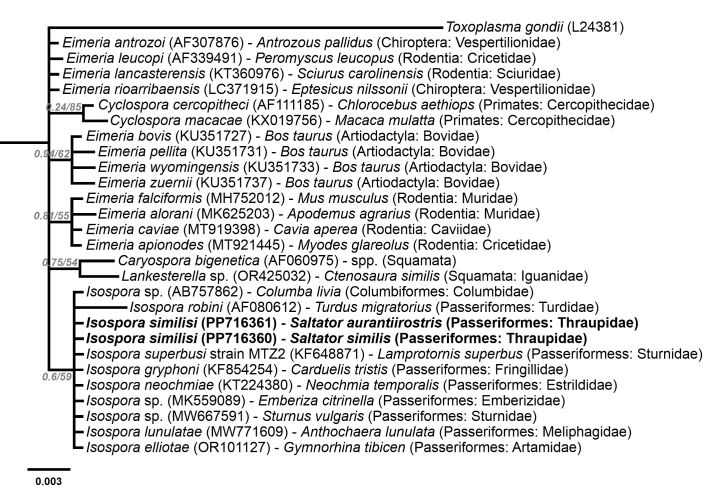
Phylogenetic relationship of *Isospora similisi* from the green-winged saltator *Saltator similis* and golden-billed saltator *Saltator aurantiirostris* inferred by Bayesian analysis for a *locus* (1NF) within 18S small subunit ribosomal RNA. Branch lengths correspond to mean posterior estimates of evolutionary distances (scale-bar: 0.003). Branch labels at the nodes show posterior probabilities under the Bayesian Inference analysis and bootstrap values derived from Maximum likelihood analysis. Only posterior probabilities higher than 0.5 are displayed. The phylograms were outgrouped using *Toxoplasma gondii* (GenBank accession number: L24381).

Phylogenetic analysis based on the MAVCOXI, MACOIII, MARI and 1NF/18S *loci* included sequences from eimeriid coccidia available in GenBank (Figures 2-5). An unnamed *Choleoeimeria* sp. (GenBank Accession No. KT203395) was used as the outgroup in the phylogenies of the mitochondrial *loci* (Figures 2-4), and *Toxoplasma gondii* (Nicolle & Manceaux, 1908) (GenBank Accession No. L24381) was used as the outgroup in the phylogenetic analysis of the 1NF/18S *locus* (Figure 5).

In the cladogram produced by the phylogenetic analysis of the MAVCOXI *locus* (Figure 2), *I. similisi* exhibited monophyly with the two *Isospora* spp. from the superb starling *L. superbus* and, in a larger monophyletic group, with other *Isospora* spp. from passerines and *Eimeria* spp. from rabbits. The cladograms of the MACOIII and MARI *loci* (Figures 3 and 4) resulted in coherent monophyletic groups for *Eimeria* spp. from Galliformes and *Eimeria* spp. from rabbits, in addition to other groups of *Isospora* spp. from passerines, including *I. similisi* from *S. similis* and *S. aurantiirostris*. Lastly, the phylogenetic analysis of the 1NF/18S *locus* (Figure 5) resulted in coherent monophyletic groups for *Cyclospora* spp., *Eimeria* spp. from cattle, *Eimeria* spp. from rodents, *Isospora* spp. from passerines and *Lankesterella* sp. and *Caryospora* sp., which are related genera, in addition to *Eimeria* spp. from bats and squirrels that were not monophyletically grouped.

## Discussion

**Figure 6:**
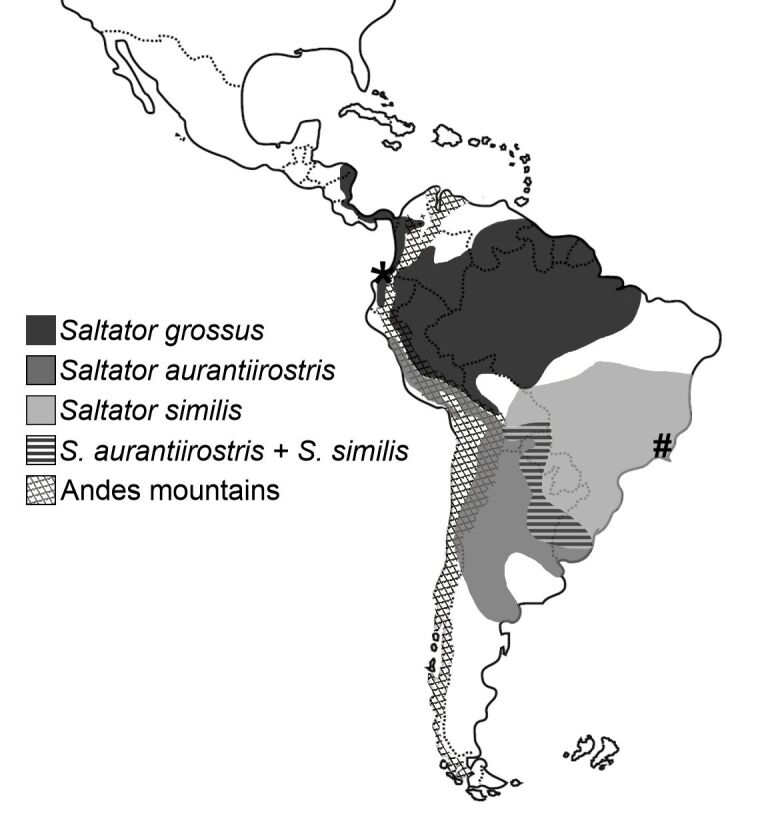
Geographic range of several *Saltator* spp. in the Neotropical region (based on data from BirdLife International (2024)). The type host of *Isospora formarum* is the slate-colored grosbeak *Saltator grossus*, and the type host of *Isospora similisi* is the green-winged saltator *Saltator similis*. The new host for *I. similisi* is the golden-billed saltator *Saltator aurantiirostris*. *Saltator grossus* has trans-Andean and cis-Andean populations. *Saltator similis* and *S. aurantiirostris* are sympatric with each other in south-central South America, but both are allopatric with *S. grossus*. The asterisk and sharp indicate approximately the type localities of *I. formarum* (trans-Andean) and *I. similisi* (cis-Andean), respectively.

Wild birds are recognized as bioindicators of environmental conservation, since the presence of certain specialist species and/or those with high forest dependence dictates preserved/conserved environments. Coccidians from wild birds, in turn, can be considered biomarkers, as the increase in their densities and/or clinical signs in wild birds determine early changes in the environment (Berto & Lopes, 2020; Maronezi et al., 2024). This association is easily observed in captive birds or birds rescued from wildlife trafficking, which often show very high densities of coccidians associated with severe coccidiosis (Coelho et al., 2012, 2013; Vasconcellos et al., 2013; Maronezi et al., 2022; Berto & Lopes, 2020). In this context, Maronezi et al. (2022) highlight the risk of captivity of wild birds around conservation units, such as Itatiaia National Park, since captive birds shed high densities of coccidian oocysts in their feces, which can easily be transmitted directly or indirectly to free-living birds. Subsequently, Maronezi et al. (2024) highlighted this possibility in the first report of a free-living green-winged saltator in Itatiaia National Park showing clinical signs of coccidiosis and high density of coccidian oocysts. Lastly, the current study reports a golden-billed saltator living in captivity in the proximities of Itatiaia National Park as a new host for one of the three *Isospora* spp. recorded from green-winged saltators, thus expanding the host specificity of *I. similisi*, and consequently, its distribution in the Neotropical region (Figure 6) (BirdLife International, 2024).

The golden-billed saltator *S. aurantiirostris* is naturally distributed throughout the Chaco region, Pampas, and central portion of the Andes in the Neotropical region, through Argentina, Bolivia, Brazil, Chile, Paraguay, Peru and Uruguay. In Brazil, it is only observed in Rio Grande do Sul and southwestern Mato Grosso do Sul (Figure 6) (BirdLife International, 2024). The captivity site of the golden-billed saltator of the current study is located in southeastern Brazil; in other words, quite far from its natural area of geographic distribution (Figure 6). This finding therefore highlights the importance of the anthropomorphic dispersion of coccidians (Berto & Lopes, 2020), i.e., the role of trafficking, sale and breeding of wild birds in the transmission of coccidia among allopatric birds. Furthermore, the possibility of transmission between captive birds or from them to susceptible free-living birds is exponentially increased considering the high density of coccidian oocysts in these birds; in other words, in addition to trafficking and captivity enabling transmissions that are very unlikely, if not impossible, under natural conditions, the large numbers of oocysts shed by these birds under these conditions intensifies the successful transmission/dispersion of coccidians (Berto & Lopes, 2020; Maronezi et al., 2022, 2024). Accordingly, the parasite density in the captive golden-billed saltator of the current study was very high, although it appeared to be healthy and showed no observable clinical signs of coccidiosis. In this regard, this finding reveals the potential for transmission and dispersion of *I. similisi* by this *S. aurantiirostris* specimen.

The maximum number of 9,000 oocysts per fecal droplet from the golden-billed saltator was even greater than the maximum number (3,668) obtained from *S. similis* recovered from illegal trafficking and kept in quarantine in a wild animal rehabilitation center, where the highest densities are often observed in weakened birds (Coelho et al., 2013). It is possible that *Saltator aurantiirostris* is an evolutionarily more recent host species, which is in an early period of parasite-host adaptation (Odum, 1998; Gardner & Duszynski, 1990; Berto & Lopes, 2020). On the other hand, perhaps this specimen is undergoing a primary infection where the absence of immunological memory allows many merogonies and gametogonies with the generation and shedding of a large number of oocysts (Krautwald-Junghanns et al., 2009; Merino, 2010). Additionally, this high density of oocysts associated with the absence of observable clinical signs leads to the assumption that *I. similisi*, which was the only species or at least the majority species observed in fecal droplets, has very low pathogenicity. This assumption, in association with the clinical signs observed in the juvenile *S. similis* positive for the three *Isospora* spp. reported in Maronezi et al. (2024), indicates that *Isospora saltatori* Berto, Balthazar, Flausino, Lopes, 2008, more abundant in this report, and that *Isospora trincaferri* Berto, Balthazar, Flausino, Lopes, 2008, should be more pathogenic (causing severe disease). Be that as it may, these occasional findings of coccidiosis and/or high density of coccidian oocysts in a single or several specimens of *Saltator* spp. do not allow for definitive conclusions.

Five *Isospora* spp. are recorded to date from *Saltator* spp. (Maronezi et al., 2022). *Isospora pityli* McQuistion & Capparella, 1992 and *Isospora formarum* McQuistion & Capparella, 1992 were the first two species described from the slate-colored grosbeak *Saltator grossus* (Linnaeus, 1766) in the Esmeraldas Province of Ecuador (Figure 6). These two *Isospora* spp. have not been reported since their original descriptions by McQuistion & Capparella (1992). The other three *Isospora* spp. from *Saltator* spp. were identified in *S. similis* living in captivity or in a rehabilitation center in southeastern Brazil, and were later reported in other locations, also in southeastern Brazil, collected from captive and free-living birds, including from the buff-throated saltator *S. maximus* infected with *I. trincaferri* (Coelho et al., 2013; Lopes et al., 2013; Maronezi et al., 2022, 2024).

As previously pointed out by Maronezi et al. (2022), *I. pityli* and *I. formarum* are morphologically similar to *I. saltatori* and *I. similisi*, respectively, with the exception of some minor differences in a few taxonomic features. In this regard, the main criterion for differentiating these *Isospora* spp. is ecological, since *I. pityli* and *I. formarum* were described in Ecuador, in the trans-Andean region; while *I. saltatori* and *I. similisi* have so far been reported in southeastern Brazil, in the cis-Andean region (Figure 6). In other words, in a conservative approach, the strong geographic barrier of the Andes mountain range that separates the locations where these *Isospora* spp. have been reported and described precludes the establishment of junior synonyms for these species. On the other hand, the wide geographic distribution of *Saltator* spp., such as *S. grossus* which has cis-Andean and trans-Andean populations, in addition to the distribution of *S. similis* and *S. aurantiirostris*, the latter being a new host for *I. similisi* in the current study, enables the distribution and dispersion of their coccidian parasites throughout most of the Neotropical region (Figure 6) (Berto & Lopes, 2020). It is worth mentioning that *S. similis* and *S. aurantiirostris* are not sympatric with *S. grossus*, although the distribution of *S. aurantiirostris* borders the cis-Andean population of *S. grossus* in the Andes mountains and the distribution of *S. similis* borders that of *S. grossus* in central-west Brazil (Figure 6).

In this context, the findings of the current study reinforce the possibility of *I. similisi* being established as a junior synonym of *I. formarum*. Coccidia dispersion across the Andes mountains, facilitated by the natural distribution and dispersion of their hosts, has been made in the identification of *Isospora sagittulae* McQuistion & Capparella, 1992 from antbirds (Berto et al., 2014b; Silva-Carvalho et al., 2018) and *Isospora bellicosa* Upton, Stamper & Whitaker, 1995 from icterid birds (Silva et al., 2017). In contrast, the current study only demonstrated the susceptibility of *S. aurantiirostris* to *I. similisi* in captivity, albeit without identifying it from free-living golden-billed saltators in their natural distribution area. In a conservative approach, this does not favor the establishment of *I. similisi* as a junior synonym of *I. formarum*. The ideal strategy for this purpose would be the morphological and molecular identification of oocysts of *I. pityli* and *I. formarum* from their type host *S. grossus* in Ecuador, in direct comparison with oocysts identified as *I. saltatori* and *I. similisi* from *S. similis* in southeastern Brazil. In addition, the finding of these *Isospora* spp. from the buff-throated saltator *S. maximus*, which is one of the *Saltator* spp. with the widest distribution in the Neotropical realm from the trans-Andean region to the Atlantic coast of South America, would confirm this assumption of coccidia dispersion across trans- and cis-Andean saltators.

The molecular analyses performed in the current study supplemented the molecular characterization of *I. similisi*, and confirmed the finding of this coccidia species from the new host *S. aurantiirostris*. The very slight differences in sequences of *I. similisi* from *S. similis* and *S. aurantiirostris* observed in the MACOIII (2 nucleotides) and MARI (1 nucleotide) *loci* should be considered as intraspecific and possibly resulting from adaptation to another host (Berto & Lopes, 2020; Ortúzar-Ferreira et al., 2023). This conclusion can be explained by the fact that these differences compared to other *Isospora* spp. from passerines deposited in GenBank are considerably higher, in addition to these exhibiting monophyly in the phylogenetic analyses for these *loci* (Figures 3 and 4).

Mitochondrial genes are known to be less conserved, and therefore the most suitable for delimiting species, as they indicate even intra-specific differences (Ogedengbe et al., 2011; El-Sherry et al., 2013; Ortúzar-Ferreira et al., 2023). Phylogenetic analyses of mitochondrial *loci* resulted in monophyletic groups with good support of maximum likelihood bootstrap values and Bayesian posterior probabilities, mainly for *Eimeria* spp. from rabbits and *Eimeria* spp. from Galliformes and some *Isospora* spp. from passerines. However, other *Isospora* spp. from passerines were ungrouped, or incongruously grouped, in the cladograms of the three mitochondrial *loci* (Figures 2-4). The significant genotypic variations that were expected for mitochondrial genes, or some other unknown factor for *Isospora* spp. from passerines, may be associated with the incongruity of these phylogenetic results. The same observations were described by Ogedengbe et al. (2018), who demonstrated the paraphyly of *Isospora* spp. and *Eimeria* spp. from different host taxa in a comprehensive phylogenetic analysis involving both mitochondrial and 18S genes. Based on their findings, Ogedengbe et al. (2018) concluded that the necessary taxonomic adjustments aiming at a congruence of phenotypes with the genotypes of Eimeriidae would require the creation of at least 9 new genera not based on the proportion of sporocysts and sporozoites upon which coccidia taxonomy is traditionally based.

The *S. similis* and *S. aurantiirostris* samples showed identical the sequences for the 1NF/18S *locus*. In fact, the 18S gene is known to be more conserved than mitochondrial genes, and hence more suitable for the phylogenetic reconstruction of genera or higher taxa (Ogedengbe et al., 2011; El-Sherry et al., 2013; Andrade et al., 2024). A comparative molecular analysis of the 1NF/18S *locus* highlights this statement, as very small differences are observed between different coccidia species from large host taxa, including *Isospora* spp. from different orders of Aves that were 99.7% similar. Along the same vein, the phylogenetic analysis (Figure 5) showed congruent and well-defined monophyletic groups for higher taxa, such as the different genera of Eimeriidae, which were grouped in separate clades. However, *Isospora* spp., evolutionarily distant because it parasitizes different higher host taxa, belongs to the same monophyletic group.

In conclusion, after analysis and discussion of all the results obtained in the current study, the golden-billed saltator *Saltator aurantiirostris* is recorded as a new host susceptible to *I. similisi*, whose molecular characterization is supplemented by the sequencing of three mitochondrial *loci* and one nuclear *locus*. In addition, *I. similisi* is estimated to be a junior synonym of *I. formarum* due to the broad distribution of its hosts in the Neotropical region. Therefore, the findings of the current study are expected to motivate future taxonomic studies of *Isospora* spp. from other *Saltator* spp. in order to establish the synonymization of *I. formarum* with *I. similisi*, and thus, its wide distribution and dispersion in the Neotropical region, including across the Andes mountain range.
